# Response to [Time zero alignment in target trial emulation of VMAT2 inhibitors versus anticholinergics for tardive dyskinesia]

**DOI:** 10.1111/pcn.70095

**Published:** 2026-06-17

**Authors:** Tien‐Wei Hsu, Chia‐Kuang Tsai, Chih‐Sung Liang

**Affiliations:** ^1^ Department of Psychiatry E‐DA Dachang Hospital, I‐Shou University Kaohsiung Taiwan; ^2^ Department of Psychiatry E‐DA Hospital, I‐Shou University Kaohsiung Taiwan; ^3^ School of Medicine, College of Medicine I‐Shou University Kaohsiung Taiwan; ^4^ Department of Neurology, Tri‐Service General Hospital National Defense Medical University Taipei Taiwan; ^5^ Department of Psychiatry, Tri‐Service General Hospital National Defense Medical University Taipei Taiwan; ^6^ Department of Psychiatry Beitou Branch, Tri‐Service General Hospital Taipei Taiwan

## Abstract

This article relates to Time zero alignment in target trial emulation of VMAT2 inhibitors versus anticholinergics for tardive dyskinesia.

We thank the authors for raising this important point regarding time‐zero alignment.^1^ In our original design,^2^ both TD diagnosis and treatment initiation were restricted to the same contemporary study period between January 1, 2019 and June 30, 2025. This window was chosen to ensure that both VMAT2 inhibitors approved for TD in the United States were available in routine practice (valbenazine received FDA approval on April 11, 2017, and deutetrabenazine on August 30, 2017) while capturing contemporary prescribing and follow‐up. During this period, anticholinergic agents were not recommended as evidence‐based TD‐directed treatment,^3^ although they may still be prescribed in patients with TD because of coexisting or suspected extrapyramidal symptoms, such as drug‐induced parkinsonism or acute dystonia. Additionally, this restriction was intended to improve comparability between VMAT2 inhibitor and anticholinergic initiators under similar treatment availability and prescribing practice.

To further address this concern, we additionally required treatment initiation to occur after TD diagnosis. This restriction resulted in minimal change in sample size, and the estimated hazard ratios remained essentially unchanged. We appreciate the authors' thoughtful comment, which helped us improve the clarity of our study design.

## Disclosure statement

The authors have no conflict of interest to declare.

## Data Availability

Data sharing not applicable to this article as no datasets were generated or analysed during the current study.
